# Smartphone-Based Pedestrian Dead Reckoning for 3D Indoor Positioning

**DOI:** 10.3390/s21248180

**Published:** 2021-12-08

**Authors:** Jijun Geng, Linyuan Xia, Jingchao Xia, Qianxia Li, Hongyu Zhu, Yuezhen Cai

**Affiliations:** 1 Guangdong Provincial Key Laboratory of Urbanization and Geo-Simulation, School of Geography and Planning, Sun Yat-sen University, 135 # Xingangxi Road, Guangzhou 510275, China; gengjj@mail2.sysu.edu.cn (J.G.); liqianx2@mail2.sysu.edu.cn (Q.L.); zhuhy35@mail2.sysu.edu.cn (H.Z.); caiyzh5@mail2.sysu.edu.cn (Y.C.); 2 School of Civil Engineering, Guangzhou University, Guangzhou 510006, China; jcxia@gzhu.edu.cn

**Keywords:** indoor localization, 3D indoor positioning method, robust adaptive cubature Kalman filter, robust adaptive Kalman filter, 16-wind rose map

## Abstract

Indoor localization based on pedestrian dead reckoning (PDR) is drawing more and more attention of researchers in location-based services (LBS). The demand for indoor localization has grown rapidly using a smartphone. This paper proposes a 3D indoor positioning method based on the micro-electro-mechanical systems (MEMS) sensors of the smartphone. A quaternion-based robust adaptive cubature Kalman filter (RACKF) algorithm is proposed to estimate the heading of pedestrians based on magnetic, angular rate, and gravity (MARG) sensors. Then, the pedestrian behavior patterns are distinguished by detecting the changes of pitch angle, total accelerometer and barometer values of the smartphone in the duration of effective step frequency. According to the geometric information of the building stairs, the step length of pedestrians and the height difference of each step can be obtained when pedestrians go up and downstairs. Combined with the differential barometric altimetry method, the optimal height can be computed by the robust adaptive Kalman filter (RAKF) algorithm. Moreover, the heading and step length of each step are optimized by the Kalman filter to reduce positioning error. In addition, based on the indoor map vector information, this paper proposes a heading calculation strategy of the 16-wind rose map to improve the pedestrian positioning accuracy and reduce the accumulation error. Pedestrian plane coordinates can be solved based on the Pedestrian Dead-Reckoning (PDR). Finally, combining pedestrian plane coordinates and height, the three-dimensional positioning coordinates of indoor pedestrians are obtained. The proposed algorithm is verified by actual measurement examples. The experimental verification was carried out in a multi-story indoor environment. The results show that the Root Mean Squared Error (RMSE) of location errors is 1.04–1.65 m by using the proposed algorithm for three participants. Furthermore, the RMSE of height estimation errors is 0.17–0.27 m for three participants, which meets the demand of personal intelligent user terminal for location service. Moreover, the height parameter enables users to perceive the floor information.

## 1. Introduction

Recently, location-based services (LBS) have become increasingly popular in indoor environments [1]. With the rapid development of positioning technology, LBS has become an indispensable part of people’s lives. Global Navigation Satellite System (GNSS) provides accurate location services in the outdoor environment. However, due to the limitations of satellite signals, the accuracy of GNSS is degraded in indoor environments [2,3]. Therefore, additional positioning technology is needed to enhance indoor positioning. Although a lot of approaches including WiFi, Bluetooth, Ultra-Wideband (UWB), radio-frequency identification (RFID), etc. are feasible in terms of localization accuracy, the implementation of most existing localization systems is based on infrastructure which is often difficult due to the requirement of additional infrastructure such as wireless APs, pertained database, map information, foot-mounted inertial sensors, etc. [2]. We believe any realistic and generalized indoor localization system should be freed from this fundamental restriction [4]. So there are still many challenges for indoor positioning using smartphones [5,6]. The Micro-Electro-Mechanical System (MEMS) is more competitive due to its independence from the existing infrastructure in the indoor environment. This is especially important for indoor positioning because other indoor positioning technologies require additional installation or body attachment of specific sensors [5]. So an actual indoor pedestrian tracking method should consider the indoor environment, without a pre-trained database or graphic, which is the key to developing an effective indoor positioning system. In addition, MEMS sensors are embedded in each smartphone, so the sensor data of smartphones can be used to conveniently estimate the location of pedestrians. Therefore, Pedestrian Dead Reckoning (PDR) based on smartphones is more practical than other positioning methods [7]. The PDR method has become the focus of future research. Normally, people spend most of their time in indoor environments, and indoor pedestrian tracking has become a key technology required for indoor LBS in the smartphone application market [2,7]. Therefore, the realization of self-contained, stand-alone localization is the main goal of our research. To solve the above problems, the PDR method based on smartphone MEMS is a good candidate for localization since smartphones are carrying various MEMS sensors which can be used to estimate the current location [2]. This approach can be used anywhere and at any time without the need for huge infrastructure to obtain the map database in advance. However, the available sensors still have the problem of inaccurate sensing ability and limited resources.

At present, the research on the PDR method has become a hot topic, but there is still a lack of comprehensive evaluation. PDR method provides an indoor positioning method including three aspects: heading estimation, stride detection and step length estimation [7,8]. Due to the inaccurate estimation of heading and step length, the error of PDR positioning will increase over time. Therefore, the estimation of heading is one of the key issues of PDR positioning. Numerous studies are dedicated to pedestrian dead reckoning (PDR) with MARG sensors of the smartphone [3]. The progress of MEMS technology makes Magnetic Angular Rate Gravity (MARG) sensors more and more accurate, lightweight and low cost, which greatly facilitates their use in indoor positioning [9]. A smartphone-based pedestrian dead reckoning that tracks indoor users’ location is based on the use of the accelerometer, magnetometer, and gyroscope in a smartphone. Since the gyroscope suffers from accumulated measurement errors, it is not effective for measuring the angles over a long period of time [4]. In order to obtain a stable and reliable heading, the accelerometer and magnetometer can be combined with a gyroscope [3]. In general, the complementary filter (CF) and the Kalman filter (KF) are common data fusion methods [7]. Although the computational cost of CF is low and the process is simple, the accuracy of heading obtained by CF is lower than that of KF, and the results of CF are worse in dynamic environments [3,7]. KF is suitable for linear and discrete systems [10]. When the noise is white noise, the result of the Kalman filter is good. However, when the noise is colored noise or the system is uncertain, the results of Kalman filtering are suboptimal and unstable. To solve the nonlinear problem, extended Kalman filter (EKF) [11], unscented Kalman filter (UKF) [12] and cubature Kalman filter (CKF) [13,14] are proposed. Compared with EKF, CKF can avoid linearization of the nonlinear system by using cubature point sets to approximate the mean and variance [15]. Moreover, CKF has a strict mathematical derivation and has stronger adaptability than UKF [15,16]. Obviously, CKF is a good method to deal with nonlinear estimation problems. Similar to KF, CKF can obtain good performance when the complete and accurate information of noise distribution has to be known as a prior [13,15]. However, in practical applications, prior noise statistics are usually unknown or time-varying. To solve these problems, many researchers have proposed the robust filter algorithm and the adaptive filter algorithm [3,7,13,15,16]. However, there is still a lack of comprehensive evaluation. Considering robust and adaptive algorithms comprehensively, a quaternion-based robust adaptive cubature Kalman filter (RACKF) is proposed to estimate the heading of pedestrians based on magnetic, angular rate, and gravity (MARG) sensors. Based on the fading memory weighted method and the limited memory weighted method, the model noise parameters are adaptively corrected. Moreover, to control the outlier influence, the paper used a robust parameter to reduce the effects of observation outliers on positioning accuracy. A maximum likelihood-type estimator (M-estimator)-based model is used in the robust parameter to identify and control measurement outliers. In addition, this paper constructs an adaptive factor based on prediction residual to overcome the influence of filtering model error and abnormal disturbance. Currently, the traditional heading estimation usually takes the heading angle at the peak (valley) time as the heading at the current time in the PDR method [17]. In order to improve the reliability of the heading angle and weaken the randomness, this paper improves the selection method of heading angle in each step of PDR positioning. Normally, when a person walks normally, there is little difference in step size and heading between the previous step and the next step. Therefore, the step size and heading of the previous step can be used as the prior estimation of the step size and heading of the next step, and the Kalman filter is used to estimate the prior estimates and the output results of the estimation algorithm, which reduces the estimation error. In addition, based on the indoor map vector information, this paper proposes a calculation strategy of the 16-wind rose map to further improve pedestrian positioning accuracy and reduce the accumulation error for the heading angle.

The traditional pedestrian dead reckoning can achieve two-dimensional positioning, and the current research on three-dimensional pedestrian dead reckoning is relatively limited [18]. At present, the research on smartphone positioning is mostly focused on two-dimensional location. However, in the indoor environment, the LBS based on the smartphone often needs not only the two-dimensional location but also the height information. In addition, in the three-dimensional indoor positioning research, the height coordinates are mostly calculated by the barometer [17,19]. However, the stability of the barometer in the indoor environment is not good, due to the influence of the temperature and environment. The barometer data collected at different times in the same location may also vary considerably. To make up for the shortcomings of unstable barometric value, this paper adopts the method of differential barometric altimetry, which needs to use the base station and take the height of the base station and the barometric value as a reference. At the same time, the relationship between pedestrian trajectory and three-dimensional position needs to be fully excavated in indoor three-dimensional positioning. To perceive the height change of pedestrians, combined with the accelerometer, elevation angle and barometric value of the smartphone, the step frequency detection method that the step height is taken as the height change value of each step is used to calculate the height value. When going upstairs and downstairs, there are always mutations in the pitch angle and in the, total accelerometer of the smartphone, which can be used to distinguish the moving mode and improve the ability to distinguish the mode. According to many experiments, three kinds of walking motion modes are distinguished. Combined with the differential barometric altimetry and step frequency detection method, the optimal height solution can be computed by the robust adaptive Kalman filter (RAKF) algorithm. In RAKF algorithm, to overcome the filter model error and the influence of abnormal disturbance, an adaptive factor is applied by using state discrepancy statistics to overcome the abnormal influence of state disturbance. Moreover, an M-estimator-based robust estimation of the equivalent weight matrix of the measurements is used to weaken the negative impacts from measurement outliers and state model errors. 

As a result, to improve the tracking performances of pedestrian indoor positioning, this paper proposes a 3D indoor positioning method based on the built-in MEMS sensors of the smartphone. A robust adaptive cubature Kalman filtering based on the observability of the parameters is used to estimate the heading of the pedestrian. Moreover, the step length estimation can be calculated by the outputs of the accelerometer. The optimal step length and heading can be calculated by the KF. A 16-wind rose map calculation strategy is used to further optimize the pedestrian heading. Moreover, the pitch angle, total accelerometer and barometer of the smartphone are used to distinguish different types of behavior patterns. According to the characteristics of each behavior pattern, the pedestrian three-dimensional position is calculated for each effective step. When pedestrians walk, not only the barometer is used to measure height, but also the changes of pitch angle, accelerometer and barometer are used as the judgment basis of behavior mode. At the same time, the step width of the prior building information is taken as the step length, and the step height is taken as the height change value of each step in the step frequency detection method. So the stair information and the effective step length are used to calculate the position information. Combined with the differential barometric altimetry and step frequency detection method, the optimal height solution can be computed by the robust adaptive Kalman filter (RAKF) algorithm. By extracting different types of effective strides, the three-dimensional coordinate values of pedestrians with different walking patterns are calculated to realize the three-dimensional indoor positioning of pedestrians. The proposed method can easily be used at any site because the method does not require any anchor or physical map information. Based on the movement law of pedestrians, the proposed 3D indoor positioning method can effectively reduce the influence of sensor cumulative error on position calculation, and improve the positioning accuracy. Our contributions are as follows:

Firstly, a robust adaptive cubature Kalman filter algorithm is proposed for attitude and heading estimation based on MARG sensors. Based on the fading memory weighted method and the limited memory weighted method, the model noise parameters are adaptively corrected. A robust M-estimator is used to identify and control measurement outliers and an adaptive factor is used to overcome the influence of the filter model errors and abnormal disturbances. 

Second, the heading and step length of each step are optimized by a Kalman filter to reduce positioning error. Based on the indoor map vector information, this paper proposes a calculation strategy for the heading angle of the 16-wind rose map to further improve the pedestrian positioning accuracy and reduce the accumulation error.

Finally, combined with the differential barometric altimetry and step frequency detection method, the optimal height solution can be computed by the robust adaptive Kalman filter algorithm.

The organization of this paper proceeds as follows: the proposed 3D indoor positioning method is described in detail in Section 2. Section 3 shows the experimental and results analysis. In Section 4 the shortcomings of the experiment are discussed. Section 5 concludes this paper and presents the future work.

## 2. Materials and Methods

**Figure 1:**
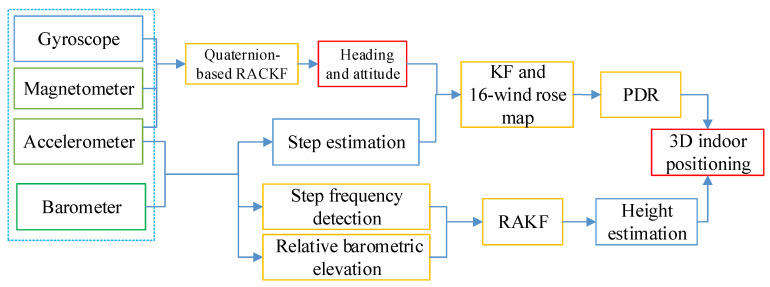
The frame of the proposed 3D indoor positioning method.

A proposed 3D indoor positioning method is used to estimate the three-dimensional coordinate of pedestrians, which uses the MEMS sensors (accelerometer, gyroscope, magnetometer and barometer) of a smartphone. As Figure 1 presents, to estimate the user’s location, quaternion-based RACKF is proposed to calculate the pedestrian heading with the outputs from the accelerometer, magnetometer and gyroscope. Moreover, the step length estimation can be calculated by the outputs of the accelerometer. The optimal step length and heading can be calculated by the KF. Finally, a 16-wind rose map calculation strategy is used to correct the pedestrian heading. When going upstairs and downstairs, the step frequency detection method is used to calculate the height variation of each step based on the geometric information of the building stairs. The barometer data in the smartphone are used to obtain the height by using the differential barometric altimetry model. Combined with the differential barometric altimetry and step frequency detection method, the two kinds of calculations are then fused using a RAKF algorithm. Then the 3D location track can be achieved. The proposed method utilizes the motion law of pedestrians, effectively reduces the influence of sensor cumulative error on position calculation, and improves the positioning accuracy.

### 2.1. Attitude and Heading Estimation Based on Quaternion-Based RACKF Algorithm

#### 2.1.1. Attitude and Heading Estimation with the Quaternion

There are several parameterizations that can represent attitude and heading, such as Euler angle, Rodrigues parameter and quaternion, etc. Since quaternion has the advantages of less computation burden and global non-singularity, quaternion has been used widely. Generally, quaternion has four elements:

(1) $$q\left(q_{0},q_{1},q_{2},q_{3}\right)=q_{0}+q_{1}i+q_{2}j+q_{3}k$$

where $q_{0}$, $q_{1}$, $q_{2}$ and $q_{3}$ are real numbers, and i, j and k are unit vectors. 

The matrix $C_{b}^{n}$ that is the coordinate transformation matrix from the ***b*** coordinate system to ***n*** coordinate system is used to calculate the heading and attitude angle, which can be described as [18]:

(2) $$C_{b}^{n}=\left[\begin{array}{ccc} q_{0}^{2}+q_{1}^{2}-q_{2}^{2}-q_{3}^{2} & 2\left(q_{1}q_{2}-q_{0}q_{3}\right) & 2\left(q_{1}q_{3}+q_{0}q_{2}\right)\\ 2\left(q_{1}q_{2}+q_{0}q_{3}\right) & q_{0}^{2}-q_{1}^{2}+q_{2}^{2}-q_{3}^{2} & 2\left(q_{2}q_{3}-q_{0}q_{1}\right)\\ 2\left(q_{1}q_{3}-q_{0}q_{2}\right) & 2\left(q_{2}q_{3}+q_{0}q_{1}\right) & q_{0}^{2}-q_{1}^{2}-q_{2}^{2}+q_{3}^{2} \end{array}\right]$$

In addition, in this paper, the axes of the navigation coordinate system point to east, north and up respectively. The matrix $C_{n}^{b}$ that is the coordinate transformation matrix from ***n*** coordinate system to the ***b*** coordinate system can be shown in the following [3,7]:

(3) $$C_{n}^{b}=\left[\begin{array}{ccc} cos\varphi cos\psi +sin\varphi sin\psi sin\theta & -cos\varphi sin\psi +sin\varphi cos\psi sin\theta & -\sin\varphi \cos\theta \\ sin\psi cos\theta & cos\psi cos\theta & sin\theta \\ sin\varphi \;cos\psi -cos\varphi sin\psi sin\theta & -sin\varphi sin\psi -cos\varphi cos\psi sin\theta & \cos\varphi \cos\theta \end{array}\right]$$

where ψ is yaw angle; φ is pitch angle; θ is roll angle.

Based on the Equations (2) and (3), Euler angles can be expressed with the quaternion as the follow [3,7]:

(4) $$\begin{array}{c} \theta =\arcsin\left[2\left(q_{2}q_{3}+q_{0}q_{1}\right)\right]\\ \varphi =\arctan\left[-\frac{2\left(q_{1}q_{3}-q_{0}q_{2}\right)}{1-q_{1}{}^{2}-q_{2}{}^{2}+q_{3}{}^{2}}\right]\\ \psi_{m}=\arctan\left[\frac{2\left(q_{1}q_{2}-q_{0}q_{3}\right)}{1-q_{1}{}^{2}+q_{2}{}^{2}-q_{3}{}^{2}}\right] \end{array}$$

The heading ψ can be obtained by the yaw $\psi_{m}$:

(5) $$\psi =\psi_{m}+D$$

where D is the local declination angle.

#### 2.1.2. Robust Adaptive Cubature Kalman Filter

Each gyroscope and magnetometer have limitations for heading estimation [18]. With time the Gyro bias increases, it results in relative azimuth drift [19,20]. In quasi-static conditions or a magnetically clean environment, the heading can be calculated based on the measured geomagnetic field [3,7]. However, single source magnetometer data cannot be used as heading information in harsh environments, especially indoors [21]. Therefore, sensor fusion is the subtle process to draw back the limitation of one sensor by another [7,19]. This paper proposes a robust adaptive Cubature Kalman filter (RACKF) algorithm to fuse the MARG sensors data to achieve more accurate results. The process of the method is explained as follows:

1. State Equation based on Gyroscope

The quaternion $\dot{q}$ is a four-dimension vector and represents the changed attitude and heading from the previous quaternion, which can be calculated from:

(6) $$\dot{q}=\frac{1}{2}q⊗w$$

where w is the angular rate vector.

The matrix form of (6) [3]:

(7) $$\dot{q}=\frac{1}{2}M\left(w\right)q=\frac{1}{2}\left[\begin{array}{cccc} 0 & -w_{x} & -w_{y} & -w_{z}\\ w_{x} & 0 & w_{z} & -w_{y}\\ w_{y} & -w_{z} & 0 & w_{x}\\ w_{z} & w_{y} & -w_{x} & 0 \end{array}\right]\left[\begin{array}{c} q_{0}\\ q_{1}\\ q_{2}\\ q_{3} \end{array}\right]$$

where $w_{x}$,$w_{y}$,$w_{z}$ are angular rate values along X, Y, and Z axes of the device coordinate system.

We could provide the analytical solution of Equation (7), and the discrete form is [22]:

(8) $$q_{k+1}=[I\times \cos(\theta /2)+A\times dt\times \sin(\theta /2)/\theta ]q_{k}$$

where *I* is the n × n unit matrix, dt is the sampling interval, *A* is the incremental angle matrix with its form of $w_{x}$, $w_{y}$, $w_{z}$, $\theta =\sqrt{{\left(w_{x}\times d_{t}\right)}^{2}+{\left(w_{y}\times d_{t}\right)}^{2}+{\left(w_{z}\times d_{t}\right)}^{2}}$.

2. Measurement Equation based on Accelerometer and Magnetometer

From the relationship between the observation vector in the body frame and the navigation frame, we can conclude that the measurement of accelerometer and magnetometer *u* is a function of *q* = [$q_{0}\cdot q_{1}\cdot q_{2}\cdot q_{3}$] [23]:

(9) $$u=\left[\begin{array}{l} a_{x}\\ a_{y}\\ a_{z}\\ m_{x}\\ m_{y}\\ m_{z} \end{array}\right]=\left(\begin{array}{c} 2(q_{1}q_{3}-q_{0}q_{2})\\ 2(q_{2}q_{3}+q_{0}q_{1})\\ q_{0}^{2}-q_{1}^{2}-q_{2}^{2}+q_{3}^{2}\\ 2(q_{1}q_{2}+q_{0}q_{3})m_{N}+2(q_{1}q_{3}-q_{0}q_{2})m_{U}\\ (q_{0}^{2}-q_{1}^{2}+q_{2}^{2}-q_{3}^{2})m_{N}+2(q_{2}q_{3}+q_{0}q_{1})m_{U}\\ 2(q_{2}q_{3}-q_{0}q_{1})m_{N}+(q_{0}^{2}-q_{1}^{2}-q_{2}^{2}+q_{3}^{2})m_{U} \end{array}\right)$$

where $a_{x}$, $a_{y}$, $a_{z}$ represent the measurement of the accelerometer in the body coordinate system. $m_{x}$, $m_{y}$, $m_{z}$ represent the measurement of the magnetometer in the body coordinate system. $m_{N}$ and $m_{U}$ stand for the component of magnetic vector in the navigation coordinate system.

In addition, this paper tried to weaken the effect of the hard iron and scale factor with the magnetic field correction model which can be established as follows [3,7]:

(10) $$m=K\left(m^{*}+m_{0}\right)=diag\left(K_{x},K_{y},K_{z}\right)(\left[\begin{array}{c} m_{x}^{*}\\ \begin{array}{c} m_{y}^{*}\\ m_{z}^{*} \end{array} \end{array}\right]+\left[\begin{array}{c} m_{x0}\\ \begin{array}{c} m_{y0}\\ m_{z0} \end{array} \end{array}\right])$$

where $m={\left[m_{x}\;m_{y}\;m_{z}\right]}^{T}$, *K* denotes a scale transformation matrix. $m_{x}^{*}$, $m_{y}^{*}$, $m_{z}^{*}$ are the raw measurements, $m_{x0}$, $m_{y0}$, $m_{z0}$ are the biases.

Based on Equations (8) and (9), the process and observation models can be described as:

(11) $$\begin{array}{l} X_{k}=F_{k-1}X_{k-1}+w_{k-1}\\ z_{k}=h\left(X_{k}\right)+v_{k} \end{array}$$

where $X_{k}={\left[q0\;q1\;q2\;q3\;\right]}^{T}$, $F_{k-1}=I\times \cos(\vartheta /2)+A\times dt\times \sin(\vartheta /2)/\vartheta$, $z_{k}={\left[a_{x}\;a_{y}\;a_{z}\;m_{x}\;m_{y}\;m_{z}\right]}^{T}$, $h\left(X_{k}\right)=\left(\begin{array}{c} 2(q_{1}q_{3}-q_{0}q_{2})\\ 2(q_{2}q_{3}+q_{0}q_{1})\\ q_{0}^{2}-q_{1}^{2}-q_{2}^{2}+q_{3}^{2}\\ 2(q_{1}q_{2}+q_{0}q_{3})m_{N}+2(q_{1}q_{3}-q_{0}q_{2})m_{U}\\ (q_{0}^{2}-q_{1}^{2}+q_{2}^{2}-q_{3}^{2})m_{N}+2(q_{2}q_{3}+q_{0}q_{1})m_{U}\\ 2(q_{2}q_{3}-q_{0}q_{1})m_{N}+(q_{0}^{2}-q_{1}^{2}-q_{2}^{2}+q_{3}^{2})m_{U} \end{array}\right)$, $w_{k-1}$ and $v_{k}$ are the noises.

**Time Update**

At the time that the posterior density function $N({\hat{x}}_{k-1|k-1},P_{k-1|k-1})$ is known. Therefore, Cholesky factorizes thusly:

(12) $$P_{k-1|k-1}=S_{k-1|k-1}S_{k-1|k-1}^{T}$$

The cubature points $X_{i,k-1|k-1}$ can be calculated as (I = 1, 2……, m, m = 2n):

(13) $$X_{i,k-1|k-1}=S_{k-1|k-1}\xi_{i}+{\hat{x}}_{k-1|k-1}$$

where $\xi_{i}$ is Basic cubature points.

Evaluate the propagated cubature points $X_{i,k|k-1}^{*}$:

(14) $$X_{i,k|k-1}^{*}=f\left(X_{i,k-1|k-1},u_{k-1}\right)$$

where f(.) is the known function; $u_{k-1}$ is the system noise.

The state prediction ${\hat{x}}_{k|k-1}$ and the state prediction covariance $P_{k|k-1}$ can be calculated as:

(15) $${\hat{x}}_{k|k-1}=\frac{1}{m}\overset{m}{\underset{i=1}{\sum }}X_{i,k|k-1}^{*}$$

(16) $$P_{k|k-1}=\frac{1}{m}\overset{m}{\underset{i=1}{\sum }}X_{i,k|k-1}^{*}X_{i,k|k-1}^{*T}-{\hat{x}}_{k|k-1}{\hat{x}}_{k|k-1}^{T}+Q_{k-1}$$

where $Q_{k-1}$ is the system noise covariance.

Based on adaptive factors of Sage-Husa and time-varying noise statistical estimation, the state noise covariance ${\hat{Q}}_{k}$ of fading memory weighting method can be calculated [15]:

(17) $${\hat{Q}}_{k}=\left(1-d_{k}\right){\hat{Q}}_{k-1}+d_{k}[W_{k}\varepsilon_{k}\varepsilon_{k}^{T}W_{k}^{T}+P_{k|k}-(\frac{1}{2n}\overset{m}{\underset{i=1}{\sum }}X_{i,k|k-1}^{*}X_{i,k|k-1}^{*T}-{\hat{x}}_{k|k-1}{\hat{x}}_{k|k-1}^{T})]$$

where $d_{k}=(1-b)/(1-b^{k+1})$, *b* is the forgetting factor; 0.95<b<0.99 $\varepsilon_{k}$ is the filter innovation; $\varepsilon_{k}=z_{k}^{}-{\hat{z}}_{k|k-1}$; $W_{k}$ is Kalman gain, $P_{k|k}$ is the corresponding error covariance.

In the limited memory weighting method, when the pedestrian is stationary or moving, one sampling period data or the nearest step data can be used as the length of the memory window respectively. The state noise covariance ${\hat{Q}}_{k}$ of the limited memory weighting filter can be expressed as [3]:

(18) $${\hat{Q}}_{k}=b{\hat{Q}}_{k-1}+d_{w}[W_{k}\varepsilon_{k}\varepsilon_{k}^{T}W_{k}^{T}+P_{k|k}-(\frac{1}{2n}\overset{2n}{\underset{i=1}{\sum }}X_{i,k|k-1}^{*}X_{i,k|k-1}^{*T}-{\hat{x}}_{k|k-1}{\hat{x}}_{k|k-1}^{T})]+d_{w}b^{w}{\hat{Q}}_{k-w}$$

where ${\hat{Q}}_{k-w}=W_{k-w}\varepsilon_{k-w}\varepsilon_{k-w}^{T}W_{k-w}^{T}+P_{k-w|k-w}-(\frac{1}{2n}\overset{2n}{\underset{i=1}{\sum }}X_{i,k-w|k-w-1}^{*}X_{i,k-w|k-w-1}^{*T}-{\hat{x}}_{k-w|k-w-1}{\hat{x}}_{k-w|k-w-1}^{T})$; $d_{w}=\left(1-b\right)/\left(1-b^{w}\right)$; *b* is the forgetting factor.

As the limited memory weighting method requires that the state noise covariance should be known at the *k* − *w* moment, the fading memory weighting method is used to calculate the state noise covariance from the initial time to the *k* − *w* time in this paper. The state noise covariance is calculated by the limited memory weighting method from the *k* − *w* + 1 moment. The faded memory weighting method and limited memory weighted method are used to estimate and correct the model noise parameters improving the accuracy of filter estimation.

**Measurement Update**

Factorize:

(19) $$P_{k|k-1}=S_{k|k-1}S_{k|k-1}^{T}$$

Estimate the cubature points $X_{i,k|k-1}$:

(20) $$X_{i,k|k-1}=S_{k|k-1}\xi_{i}+{\hat{x}}_{k|k-1}$$

The transmission of cubature points $Z_{i,k|k-1}$ can be calculated as follows:

(21) $$Z_{i,k|k-1}=h\left(X_{i,k|k-1},v_{k}\right)$$

where h(.) is known function, $v_{k}$ is the measurement noise.

Estimate the predicted measurement ${\hat{z}}_{K|K-1}$ and the innovation covariance matrix $P_{zz,k|k-1}$:

(22) $${\hat{z}}_{K|K-1}=\frac{1}{m}\overset{m}{\underset{I=1}{\sum }}Z_{i,k|k-1}$$

(23) $$P_{zz,k|k-1}=\frac{1}{m}\overset{m}{\underset{I=1}{\sum }}Z_{i,k|k-1}Z_{i,k|k-1}^{T}-{\hat{z}}_{k|k-1}{\hat{z}}_{k|k-1}^{T}+R_{k}$$

where $R_{k}$ is the measurement noise covariance.

In actual circumstances, it is difficult for pedestrians to maintain regular motion mode [3]. So an accurate functional model is very difficult to build. Moreover, during pedestrian movement, pedestrians are vulnerable to external interference, which leads to the state model cannot reflect the real movement [24,25,26,27,28,29]. In this paper, the adaptive factor (α) based on the prediction state deviation statistics is used to overcome the influence of filtering model error and abnormal disturbance [3]. The adaptive factor using two-segment functions can be expressed as [7]:

(24) $$\partial_{k}=\{\begin{array}{ll} 1, & \Delta {\tilde{X}}_{k}\leq c0\\ \frac{c0}{\Delta {\tilde{X}}_{k}}, & \Delta {\tilde{X}}_{k}>c0 \end{array}$$

where *c*0 is a constant which can be tuned depending on the practical implementation; $\Delta {\tilde{V}}_{k}$ is the statistic of the predicted state discrepancy, defined as: $\Delta {\tilde{X}}_{k}={\left[\left\|{\tilde{X}}_{k}-{\hat{x}}_{k|k-1}\right\|/tr\left(\mathrm{cov}(\varepsilon_{k},\varepsilon_{k}{}^{T}\right)\right]}^{\frac{1}{2}}$; $\Delta {\tilde{X}}_{k}$ is a least-square estimator of the state; *tr*(·) stands for the trace of a matrix.

The adaptive factor $\partial_{k}$ is used to correct the innovation covariance matrix to weaken the influence of dynamic model error and measurement outliers. The innovation covariance matrix $P_{zz,k|k-1}^{*}$ is calculated as [3]:

(25) $$P_{zz,k|k-1}^{*}=\frac{1}{m}\overset{m}{\underset{I=1}{\sum }}Z_{i,k|k-1}Z_{i,k|k-1}^{T}-{\hat{z}}_{k|k-1}{\hat{z}}_{k|k-1}^{T}+\partial_{k}{\bar{R}}_{k}$$

In this paper, an M-estimator-based robust estimation of the equivalent weight matrix ${\bar{R}}_{k}$ is used to control the outliers in the measurements. There are several formatting methods. The Huber’s method is chosen in this paper [30]. Then, the diagonal ${\bar{r}}_{k_{ii}}$ and non-diagonal ${\bar{r}}_{k_{ij}}$ elements of ${\bar{R}}_{k}$ are calculated as [7]:

(26) $${\bar{r}}_{k_{ii}}=\{\begin{array}{ll} \frac{1}{\sigma_{ii}}, & \left|r_{k_{i}}^{\prime }\right|\leq c\\ \frac{c}{\left|r_{k_{i}}^{\prime }\right|}\cdot \frac{1}{\sigma_{ii}}, & \left|r_{k_{i}}^{\prime }\right|>c \end{array}$$

(27) $${\bar{r}}_{k_{ij}}=\{\begin{array}{cc} \frac{1}{\sigma_{ij}}, & \left|r_{k_{i}}^{\prime }\right|\leq c\;and\;\left|r_{k_{j}}^{\prime }\right|\leq c\\ \frac{c}{\max\left\{\left|r_{k_{i}}^{\prime }\right|,\left|r_{k_{j}}^{\prime }\right|\right\}}\cdot \frac{1}{\sigma_{i,j}}, & \left|r_{k_{i}}^{\prime }\right|>c\;or\;\left|r_{k_{j}}^{\prime }\right|>c \end{array}$$

where $\sigma_{ii}$ and $\sigma_{ij}$ are diagonal and non-diagonal elements of the measurement noise covariance matrix *R_k_*. *c* is a constant, and it is usually within the range of [1.3, 2.0]. $r_{k_{i}}^{\prime }$ denotes the standard residual, and it is calculated by:

(28) $$\left|r_{k_{i}}^{\prime }\right|=\left|\frac{r_{k_{i}}}{\sigma_{r_{k_{i}}}}\right|$$

where $r_{k_{i}}$ is the residual of the measurement $z_{k_{i}}$; $\sigma_{r_{k_{i}}}$ is the mean deviation of $r_{k_{i}}$.

Estimate the cross-covariance matrix:

(29) $$P_{xz,k|k-1}=\frac{1}{m}\overset{m}{\underset{I=1}{\sum }}X_{i,k|k-1}Z_{i,k|k-1}^{T}-{\hat{x}}_{k|k-1}z_{k|k-1}^{T}$$

The Kalman gain $W_{k}$ can be described:

(30) $$W_{k}=P_{zz,k|k-1}P_{xz,k|k-1}^{-1}$$

The state update ${\hat{x}}_{k|k}$ and the corresponding error covariance $P_{k|k}$ can be written as:

(31) $${\hat{x}}_{k|k}={\hat{x}}_{k|k-1}+W_{k}\left(z_{k}-{\hat{z}}_{k|k-1}\right)$$

(32) $$P_{k|k}=P_{k|k-1}-W_{k}P_{zz,k|k-1}W_{k}^{-1}$$

### 2.2. Speed Estimation

The speed estimation research mainly includes step frequency detection and step length estimation [3]. Many step detection algorithms have been proposed by researchers, including peak detection, threshold setting, zero velocity update, autocorrelation and finite-state machine (FSM) [31]. This paper uses the peak detection method to detect a step. Studies have shown that the step length is related to the acceleration, height, and strides of different people, and the step length estimated by different methods differs little [7,31]. In this paper, a nonlinear step length estimation algorithm is adopted, which takes the maximum and minimum acceleration of pedestrians within one step as the characteristic quantity. The nonlinear step length estimation model is as follows [32]:

(33) $$L_{k}=S\times \sqrt[{4}]{\left(a_{\max}-a_{\min}\right)}$$

where *L_k_* is the step length; $a_{\max}$ and $a_{\min}$ is the maximum acceleration and minimum acceleration in one step; *S* is the personalized parameter that needs to be calibrated for each pedestrian.

At present, the step frequency detection algorithm based on MEMS uses the peak detection method to find the maximum acceleration of fixed time window according to the periodic change of pedestrian acceleration. The main purpose of step frequency detection is to identify the starting point of the stride from the continuous sensor data, so as to facilitate data processing in the unit of a single step when calculating the subsequent step length and direction. When performing pedestrian step frequency detection, it is best to use the total acceleration in three directions during pedestrian walking. The numerical fluctuation of the total acceleration can reflect the human walking law to a large extent. The total acceleration can be expressed as:

(34) $$a=\sqrt{a_{x}^{2}+a_{y}^{2}+a_{z}^{2}}$$

where, $a_{y}$ and $a_{z}$ are the three directional acceleration components.

### 2.3. Height Estimation

With the rapid development of the economy and technology, the floors of many large buildings are getting higher and higher, so it is necessary to study the height measurement method of pedestrians in high-rise buildings. There are many height measurement methods, including laser altimeter, barometric, accelerometer and GPS (Global Positioning System). Since the GPS signal may not be received in a room and the laser is susceptible to complex environmental interference in a room, the two methods cannot be used for indoor height measurement. Due to the cumulative error caused by the integration of accelerometer data, an accelerometer cannot be used for indoor height measurement alone. The barometer is widely used because of its simple equipment and its accuracy can meet the requirements of certain indoor positioning. A mean filtering is applied to smooth the measured pressure of the barometer in this paper. The height value relative to the standard barometric pressure is calculated as follows: [33]:

(35) $$H_{k}=44330\times [1-{\left({\bar{p}}_{k}/p_{0}\right)}^{0.1902631}]$$

where ${\bar{p}}_{k}$ the average of atmospheric pressure, $p_{0}$ is the standard atmospheric pressure.

In practical indoor positioning applications, the areas we need to locate are often small. The closure of indoor space often makes the atmospheric environment in the vertical direction of the positioning area basically equal, and there is a state of fluid static balance. Therefore, we can use the method of differential barometric altimetry to reduce the influence of the atmospheric environment on barometric height measurement and improve the accuracy of height measurement. In daily life, people usually calculate the height of the ground floor and describe the height of the floor. Therefore, to reduce the influence of the actual environment on the measurement and ensure the readability of the measurement results, the relative height of the indoor positioning point can be calculated by taking the first floor of the indoor building as the height calculation surface. The relative elevation of the measuring point and the reference point is obtained by using the differential barometric altimetry method. The differential barometric altimetry model is as follows:

(36) $$\Delta H=H_{k}-H_{0}=4946.55\times [({\bar{p}}_{k}^{{}^{0.1902631}}-{\bar{p}}_{i}^{{}^{0.1902631}})]$$

where Δ*H* is the relative elevation from the initial elevation; ${\bar{p}}_{i}^{{}^{}}$ is the mean atmospheric pressure measured by pedestrians at the starting position of indoor positioning, and ${\bar{p}}_{k}^{{}^{}}$ is the mean atmospheric pressure measured by pedestrians when they reach position *k*.

In addition, since the pedestrian trajectory is closely related to height value, pedestrian behavior patterns can be distinguished by detecting the changes of pitch angle, accelerometer and barometer of the smartphone in the duration of effective stride frequency. In the actual situation, the barometer values always increase or decrease when pedestrians go up and downstairs. However, barometer values are volatile after a long time of movement on the same floor. Fortunately, there is a trend of continuous barometer values increasing or decreasing. The barometer value showed a decreasing trend when upstairs, and an increasing trend when downstairs. Moreover, there are always mutations in the pitch angle, total accelerometer and barometer values of the smartphone when going up and downstairs, which can be used to distinguish the moving mode and improve the ability to distinguish the mode. Therefore, the scenario of pedestrians upstairs and downstairs can be judged by the pitch angle, total accelerometer and barometer values. In addition, there is a certain degree of swing and jitter in the walking process of pedestrian-held smartphones, which will bring a small range of shake to the pitch, accelerometer information and barometer value, resulting in interference. So considering the number of steps between the floors, three continuous effective steps are selected to identify the motion mode. If there are mutations in the pitch angle, total accelerometer and barometer values in the three consecutive valid strides, the mode of going upstairs and downstairs is determined.

(37) $$\begin{array}{l} |a_{k}|>\phi_{a}\\ |\varphi_{k}|>\phi_{\varphi }\\ |\Delta h_{k}|>\phi_{h} \end{array}$$

where $a_{k}$, $\varphi_{k}$ and $\Delta h_{k}$ is the total acceleration, pitch angle and barometer values in the effective step; $\phi_{a}$, $\phi_{\varphi }$ and $\phi_{h}$ are thresholds for the total acceleration, pitch angular and barometer values for the behavior mode judgment.

The pedestrian motion state can be judged by combining the pitch angle, total accelerometer and barometer of the smartphone. In order to increase the accuracy of pedestrian motion mode judgment, this paper proposes a step frequency detection method to calculate the relative elevation information of pedestrians combined with the geometric information of the stair.

(38) $$\Delta H=vuS_{H}$$

where *v* is the behavior pattern marker; when walking in two-dimensional plane, *v* is ‘0’; when going upstairs and downstairs, *v* is ‘1’; *u* is the upstairs or downstairs marker, *u* is recorded as ‘1’, when upstairs and when downstairs, *u* is ‘−1’, $S_{H}$ is the height of one stair.

Pedestrian height variations are derived from effective step-by-step and building stair information when upstairs and downstairs. So the height is calculated by combining step frequency detection method based on the geometric information of the building stairs with the differential barometric altimetry method. Two kinds of height measurements are fused by using robust adaptive Kalman filter (RAKF) fusion. The process and observation models can be described as:

(39) $$\begin{array}{l} X_{k}=A_{k-1}X_{k-1}+w_{k-1}\\ Z_{k}=H_{k}X_{k}+v_{k} \end{array}$$

where $X_{k}={\left[\Delta h\;\;\Delta S_{h\;}\;\right]}^{T}$, $A_{k-1}=\left[\begin{array}{cc} 1 & 0\\ 0 & 1 \end{array}\;\right]$, $Z_{k}=\left[\begin{array}{l} \Delta h1\\ \Delta h2 \end{array}\right]$, $H_{k}=\left[\begin{array}{cccc} 1 & 0 & 1 & 0\\ 0 & 1 & 0 & 1 \end{array}\right]$, $w_{k-1}$ and $v_{k}$ are the noises.

The robust adaptive KF algorithm details are presented as follows:

The predicted state $X_{k|k-1}$ can be calculated as:

(40) $$X_{k|k-1}=A_{k}X_{k-1}$$

where $A_{k}=\left[\begin{array}{cc} 1 & 0\\ 0 & 1 \end{array}\;\right]$ computing the predicted state error variance matrix $P_{k|k-1}$:

(41) $$P_{k|k-1}=A_{k}P_{k-1}A_{k}^{T}+Q_{k}$$

where *Q_k_* is the state model noise covariance matrix.

In the robust adaptive Kalman filter algorithm, the gain matrix $K_{k}$ has different computations. The motion state of pedestrians is complex, so it is very difficult to construct an accurate function model. At the same time, in the process of movement, pedestrians will inevitably be affected by abnormal external interference, which means the state model cannot truly reflect the movement of pedestrians. This paper uses the adaptive factor based on state discrepancy statistics to overcome the influence of filtering model error and abnormal disturbance [3]. The adaptive factor with two-segment functions can be expressed as [7]:

(42) $$\partial_{k}=\{\begin{array}{ll} 1, & \Delta {\tilde{X}}_{k}\leq c0\\ \frac{c0}{\Delta {\tilde{X}}_{k}}, & \Delta {\tilde{X}}_{k}>\;c0 \end{array}$$

where *c*0 is a constant which can be tuned depending on the practical implementation; $\Delta {\tilde{X}}_{k}$ is the statistic of the state discrepancy statistic for judging the state model errors.

(43) $$\Delta {\tilde{X}}_{k}={\left[\left\|{\tilde{X}}_{k}-{\hat{X}}_{k|k-1}\right\|/tr\left({\hat{P}}_{k|k-1}\right)\right]}^{\frac{1}{2}}$$

where *tr*(·) stands for the trace of a matrix, ${\tilde{X}}_{k}$ is a least-square estimator of the state.

(44) $${\tilde{X}}_{k}={\left(A_{k}^{T}P_{k}A_{k}\right)}^{-1}A_{k}^{T}P_{k}Z_{k}$$

where $P_{k}$ denotes the weight matrix.

The appropriate gain matrix $K_{k}$ is obtained as:

(45) $$K_{k}=\frac{1}{\partial_{k}}P_{k|k-1}H_{k}^{T}{\left(\frac{1}{\partial_{k}}H_{k}P_{k|k-1}H_{k}^{T}+{\bar{R}}_{k}\right)}^{-1}$$

where $\partial_{k}$ is the adaptive factor, ${\bar{R}}_{k}$ is the equivalent weight matrix of the measurements.

In the measurements, this paper uses an M-estimator-based robust estimation of the equivalent weight matrix ${\bar{R}}_{k}$ to control the outliers. There are several formatting methods. The Huber’s method is chosen in this paper [30]. Then, the diagonal ${\bar{r}}_{k_{ii}}$ and non-diagonal ${\bar{r}}_{k_{ii}}$ elements of ${\bar{R}}_{k}$ are calculated as [7]:

(46) $${\bar{r}}_{k_{ii}}=\{\begin{array}{ll} \frac{1}{\sigma_{ii}}, & \left|r_{k_{i}}^{\prime }\right|\leq c\\ \frac{c}{\left|r_{k_{i}}^{\prime }\right|}\cdot \frac{1}{\sigma_{ii}}, & \left|r_{k_{i}}^{\prime }\right|>c \end{array}$$

(47) $${\bar{r}}_{k_{ij}}=\{\begin{array}{cc} \frac{1}{\sigma_{ij}}, & \left|r_{k_{i}}^{\prime }\right|\leq c\;and\;\left|r_{k_{j}}^{\prime }\right|\leq c\\ \frac{c}{\max\left\{\left|r_{k_{i}}^{\prime }\right|,\left|r_{k_{j}}^{\prime }\right|\right\}}\cdot \frac{1}{\sigma_{i,j}}, & \left|r_{k_{i}}^{\prime }\right|>c\;or\;\left|r_{k_{j}}^{\prime }\right|>c \end{array}$$

where $\sigma_{ii}$ and $\sigma_{ij}$ are diagonal and non-diagonal elements of the measurement noise covariance matrix ***R_k_***. *c* is a constant, and it is usually within the range of [1.3, 2.0]. $r_{k_{i}}^{\prime }$ denotes the standard residual, and it is calculated by:

(48) $$\left|r_{k_{i}}^{\prime }\right|=\left|\frac{r_{k_{i}}}{\sigma_{r_{k_{i}}}}\right|$$

where $r_{k_{i}}$ is the residual of the measurement $z_{k_{i}}$. And $\sigma_{r_{k_{i}}}$ is the mean deviation of $r_{k_{i}}$.

Computing the corrected state:

(49) $${\hat{X}}_{k|k}={\hat{X}}_{k|k-1}+K_{k}\left(Z_{k}-H_{k}{\hat{X}}_{k|k-1}\right)$$

Updating the state error variance matrix:

(50) $${\hat{P}}_{k|k}=(I-K_{k}H_{k}){\hat{P}}_{k|k-1}$$

### 2.4. The Proposed 3D Indoor Positioning Method

At present, the research on PDR has garnered a large amount of attention, but there is still a lack of comprehensive evaluation [34]. PDR methods on smartphones are self-contained without requiring any external infrastructures. This technology can be used anytime and anywhere, just a smartphone, without huge infrastructure. Therefore, the PDR method based on smartphones has become the focus of future research. Due to the inaccurate estimation of heading and step length, the error of PDR will increase over time especially for smartphones with cheap and noisy built-in inertial sensors. [7]. Therefore, the estimation of heading is one of the key issues of indoor positioning [3,7]. The traditional heading angle estimation is usually from the peak (valley) moment of the heading angle at the current time. To improve the reliability of the heading angle and weaken the randomness, this paper improves the selection method of direction angle in each step of PDR positioning. Since each step contains multiple heading angles at different times, the average value of the heading in one step is selected as the heading of this step to weaken the fluctuation phenomenon starting from the peak (valley) time.

In addition, when a person walks normally, the difference in step length and heading between the previous step and the next step is very small. Therefore, the step length and heading of the previous step can be used as the prior estimation of the step length and heading of the next step. Some research about context detection has already been started, however, most lacks comprehensive consideration. Based on the prior estimation and the output results of the step size and heading estimation algorithm, this paper uses KF to reduce the estimation error. The state equation and measurement equation are established as follows:

(51) $$\begin{array}{l} X_{k}=F_{k-1}X_{k-1}+w_{k-1}\\ z_{k}=H_{k}X_{k}+v_{k} \end{array}$$

where $X_{k}={\left[\psi \;L\;\right]}^{T}$, $F_{k-1}=\left[\;\begin{array}{cc} 1 & 0\\ 0 & 1 \end{array}\;\right]$, $z_{k}=\left[\begin{array}{l} \psi \\ L \end{array}\right]$, $H_{k}=\left[\begin{array}{cc} 1 & 0\\ 0 & 1 \end{array}\;\right]$, $w_{k-1}$ and $v_{k}$ are the noises, ψ is the heading, L  is the step length.

**Figure 2:**
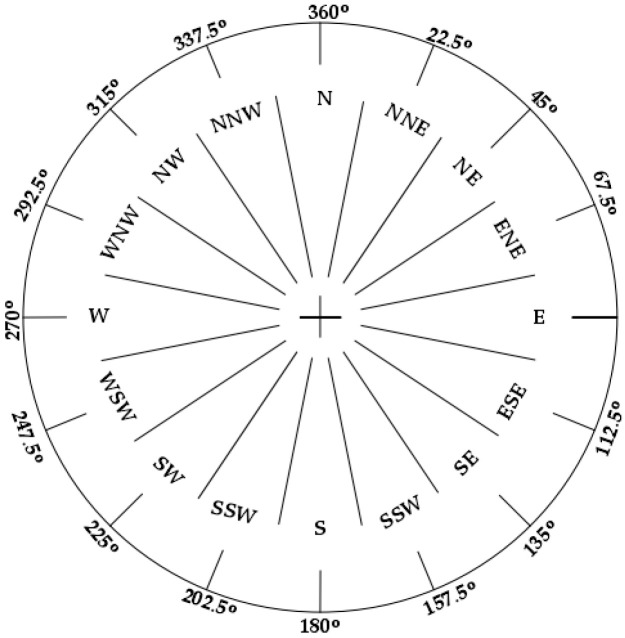
The 16-wind rose map.

To improve the estimation accuracy of heading angle and reduce the error accumulation in the PDR method, this paper proposes a heading calculation strategy of a 16-wind rose map based on the vector information of the indoor map. The general wind rose map adopts eight or sixteen directions. This paper uses 16-wind rose map to optimize heading angle estimation. Figure 2 shows a 16-wind rose map that there are 16 equidistant intervals in the wind rose map, and each interval is 22.5 degrees. When a person enters the room, there are 16 possible directions. The central direction of each interval is the direction of pedestrian movement.

In this paper, the pedestrian position is mapped to the indoor map, and 16 possible equidistant directions are virtually designed. The starting direction is the north of the map. The center direction of the nearest interval is used as the heading. If the difference between the estimated heading angle and the nearest map direction is less than 5 degrees, the estimated heading angle is used as the pedestrian’s heading, which can limit the gradual increase of heading angle error.

The conventional PDR only estimates the position in 2D space and cannot locate the pedestrian’s position in 3D space [35]. For three-dimensional indoor positioning research, the height coordinates are mostly calculated by the barometer, which is unstable. In this paper, a three-dimensional pedestrian indoor positioning algorithm is proposed. When pedestrians walk, the pitch angle, accelerometer and barometer of the smartphone are used to distinguish different types of behavior patterns including stationary, motion, upstairs and downstairs. The step width of the prior building information is taken as the step length, and the step height is taken as the vertical step length. Stair information and the effective step length are used to calculate the position information based on the step frequency detection method. Combined with the differential barometric altimetry algorithm, the robust adaptive Kalman filter is used to obtain the optimal estimation value of height. Pedestrian height calculation process reference Section 2.2. The plane coordinates of the pedestrian are calculated as follow:

(52) $$\begin{array}{l} X_{k}=X_{k-1}+vS_{w}\sin(\psi_{k})+(1-v)L_{k}\sin(\psi_{k})\\ Y_{k}=Y_{k-1}+vS_{w}\cos(\psi_{k})+(1-v)L_{k}\cos(\psi_{k}) \end{array}$$

where *k* is the current *k* effective step; $\psi_{k}$ is the heading angle of the current step; *L* is the step length; $S_{w}$ is the width of the stairs; *v* is a behavior pattern marker, marked as ‘0’ when the pedestrian is detected as two-dimensions motion and as ‘1’ when the pedestrian is detected as three-dimensional motion.

## 3. Experiments and Result Analysis

**Figure 3:**
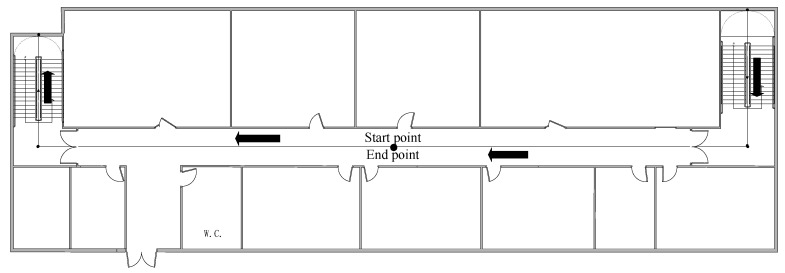
Floor plans of the site for the test.

To verify the accuracy of the proposed approach, the experiments were conducted on the sixth and seventh floors in a research building. The floor plans are presented in Figure 3. The smartphone MI 5 is selected as the test device, and its sampling frequency is 50 Hz. So the sampling frequency of data is 50 Hz based on the data acquisition software we developed. The initial state noise and measurement noise covariance matrices of the proposed filter were empirically determined depending on each measurement outputted by the smartphone in the test [3,7]. For the tests, $Q=diag(e^{-5},e^{-5},e^{-5})$, $R=diag(e^{-3},e^{-3},e^{-3})$, where diag(.) represents a diagonal matrix. Based on multiple experiments, the special parameters were empirically determined. The forgetting factor b is 0.96. To ensure that human positioning errors are detected, we only take into account the case in which the users hold their smartphone in hand, which is the most common pedestrian navigation mode. In the experiment, participants started from the midpoint of the corridor on the 7th floor of the experimental building and walked along the corridor to the 7th staircase at a normal pace. Then the participants went down to the sixth floor, continued along the corridor to the 6th floor staircase, and went up to the 7th floor. Finally, participants go back to the starting point along the corridor, as shown in Figure 3. There are three participants in the test, which can avoid the possibility of errors to a certain extent. Moreover, the experimental results of the three experimenters can be compared with each other to increase the reliability of the experimental results. Besides, because each person’s step length is different, the experimental results of three people can increase the universality of the proposed method. During the experiment, pedestrians hold equipment in hand and keep level. The length of the location traces that they walked was as long as 118 m. Participants maintained a uniform walking speed for the experiment. In the experiment, three participants of different heights and weights, as shown in Table 1. S is the parameter for establishing step size. The value of S is shown in the following table.

### 3.1. Height Experiment and Result Analysis

**Figure 4:**
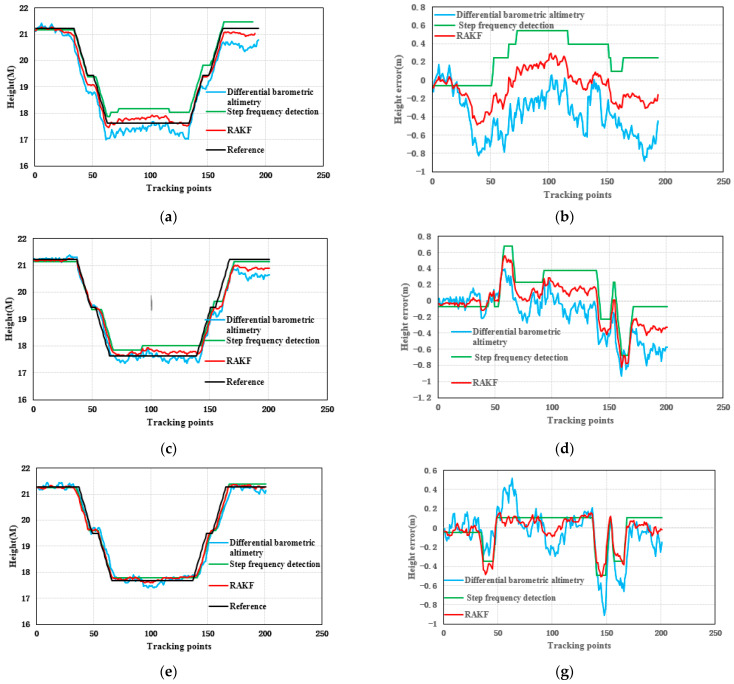
Distributions of the height test for three participants: (**a**,**b**) participant 1, (**c**,**d**) participant 2, and (**e**,**f**) participant 3.

**Figure 5:**
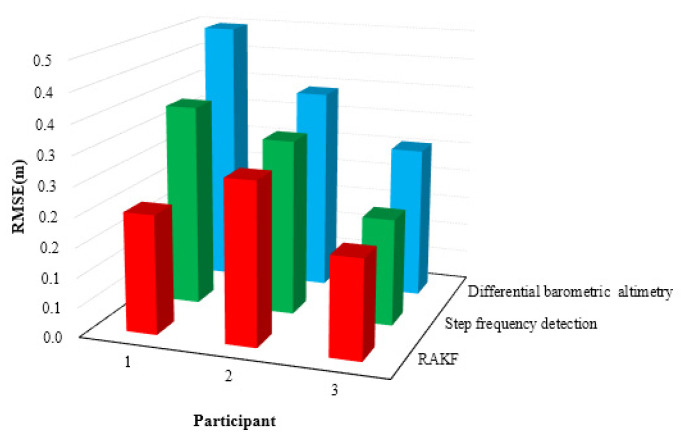
Distributions the RMSE of the height in location tracking for three participants.

The height results of the test are the relative height presented in Figure 4. Figure 4a,c,e shows the results of the height. The black line in Figure 4 is the actual reference trace. Figure 4b,d,f presents the height error. From Figure 4, we can see that the height errors of the relative elevation results calculated by the differential barometric altimetry method were larger and more unstable. This is because the barometric sensor is vulnerable to the influence of wind, humidity and temperature, which affects its measurement accuracy, resulting in unstable estimation accuracy. Although the height values computed by the step frequency detection method are stable, the step frequency detection method depends not only on the judgment of the number of stairs but also on the prior stair height, which will limit its application. The RAKF method fuses the result of differential barometric altimetry and step frequency detection method to obtain stable and reliable height. The results calculated by the robust adaptive Kalman filter (RAKF) are more stable and accurate. Table 2 and Figure 5 give the statistical results of the height errors. According to the results of Table 2, the RMSE of RAKF results were more accurate than those of differential barometric altimetry and step frequency detection method. Compared with the differential barometric altimetry and step frequency detection method, the RMSE of the height of the RAKF results decreased to about 55.23% and 41.01% respectively in the first participant test. The RMSE of the height of the second participant RAKF results decreased by 20.57% and 8.10%, and the last participant results decreased by 34.94% and 7.45% respectively.

### 3.2. 3D Indoor Positioning Experiment and Result Analysis

**Figure 6:**
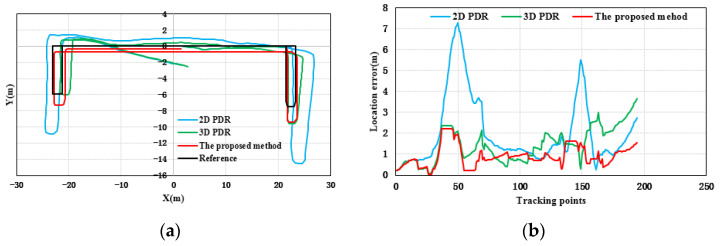

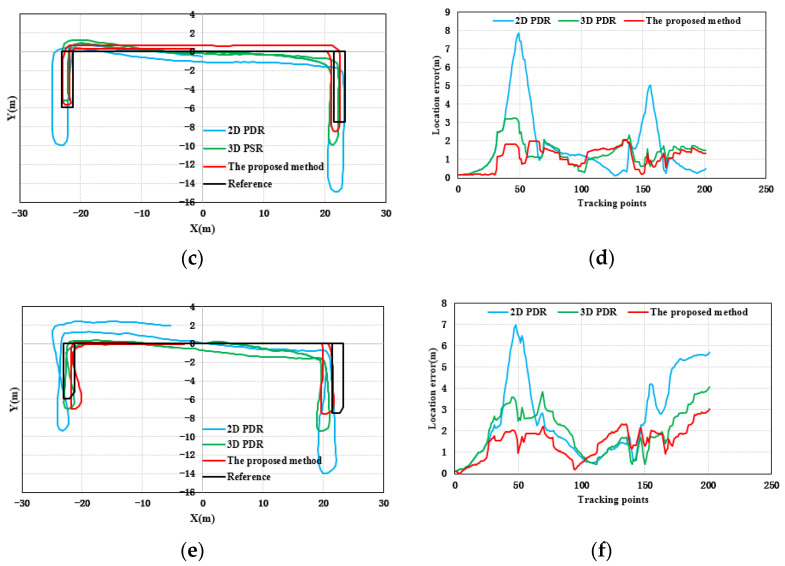
Distributions of location errors with respect to three participants, (**a**,**b**) participant 1, (**c**,**d**) participant 2, and (**e**,**f**) participant 3.

**Figure 7:**
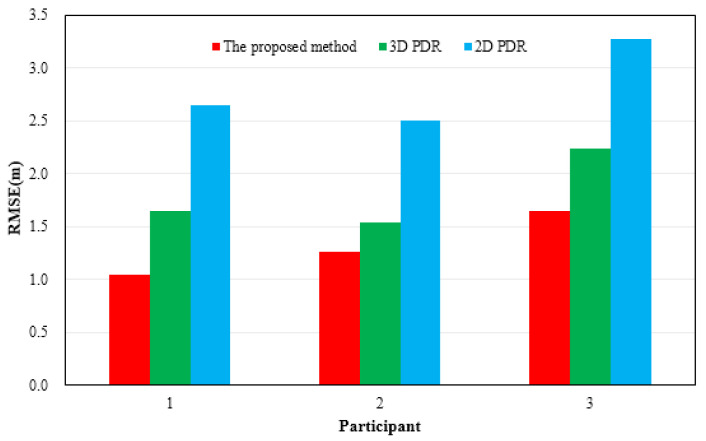
Distributions of the RMSE of location results in location tracking for three participants.

Figure 6 shows the results of location tracking, which can reflect the plane coordinates of the proposed method estimation. The black line in Figure 6 is the reference trace. Figure 6 illustrates the comparison of the location and location errors calculated by 2D PDR, 3D PDR and the proposed method. In Figure 6, for all of the three participants, compared with the results of 2D PDR, the results of 3D PDR and the proposed method approximate the reference trace better for the three participants, because the 2D PDR method didn’t take into account the change of step size when pedestrians went up and downstairs. When upstairs and downstairs, the step length is related to the width of the stair. In addition, the results of the proposed method were more accurate and stable than those of 3D PDR because the proposed method uses the average value of the heading in one step as the heading of this step to weaken the fluctuation phenomenon starting from the peak (valley) time. The Kalman filter is introduced to reduce the fluctuation of results. Moreover, a 16-wind rose map is applied to improve the estimation accuracy and solve the problem of error accumulation in the PDR to a certain extent. Table 3 and Figure 7 give the statistical results of the location errors. Compared with the 2D PDR and 3D PDR, the RMSE of location errors of the proposed method results decreased about 55.23%, and 41.01%, respectively, in the first participant test. The RMSE of location errors of the second participant results decreased by 49.28% and 17.79%, and the last participant results decreased by 49.67%, and 26.24%, respectively. 

## 4. Discussion

This paper proposed a 3D indoor positioning method based on the built-in MEMS sensors of smartphones for pedestrian positioning. Although the proposed method can realize stable and accurate positioning results, there are still some problems to discuss.

(1) Unlike most works, the proposed model is based on smart phone multi-sensor fusion to achieve indoor three-dimensional positioning. Considering filtering adaptability and robustness, a RACKF algorithm is proposed to estimate pedestrian heading. At the same time, the 16-wind rose map is introduced to improve the heading accuracy and weaken the PDR error accumulation. In addition, the accelerometer, pitch angle and barometer information are fused to identify the motion mode of pedestrians. Combined with differential barometric altimetry and step frequency detection method, this paper proposes a RAKF algorithm to estimate the relative elevation of pedestrians when upstairs and downstairs. However, the proposed method is only suitable for pedestrians holding the smart phone mode with their hands, to maintain the level. It is not suitable for phone calls, pockets and swing modes, which will be our future research work. The proposed method needs to know the height and width of the stairs in advance, which also limits the application to a certain extent. In addition, the proposed elevation estimation method is only applicable for pedestrians upstairs and downstairs, and is not suitable for elevator and escalator mode. Based on these constraints, our future work will focus on a more comprehensive positioning model. In addition, because the RACKF algorithm comprehensively considers the robustness and adaptability, the complexity of the algorithm is increased. Compared with the traditional PDR algorithm, the proposed algorithm increases the computation time. In elevation calculation, due to the integration of the results of the two algorithms, the complexity and calculation cost are increased compared with those of the traditional barometer.
(2) Due to the indoor complex environment, there were multiple sources influencing pedestrian positioning. Although the proposed 3D indoor positioning method can weaken the influence of PDR positioning error accumulation to a certain extent, the PDR positioning error is still likely to accumulate, so it is necessary to further improve the performance of the algorithm to reduce the PDR positioning error.
(3) In laboratory conditions, participants are required to walk normally, so the accuracy of pedestrian step frequency detection is high. However, in the real scenarios, the walking habits of pedestrians, the ground slope of the site and other factors, especially the conversion between walking and non-walking movements in the actual walking process, will affect the accuracy of pedestrian step frequency detection. In view of the pedestrian positioning requirements under complex walking conditions, a step frequency detection fusion algorithm with strong robustness is needed.
(4) This article considers the static, uniform movement of the state of going upstairs and downstairs, but the pedestrian movement state is varied, such as in situ walking, fast walking, slow walking, turning and so on. In a specific time window, the statistical characteristics of sensor signals, such as mean, variance, and kurtosis can be used to form feature vectors to analyze and identify different motion modes. Moreover, this paper focuses on the hand-held mode which is the most common mode for pedestrian navigation. Other carrying modes include in-pocket, swinging-hand, etc. Some methods need to be used to identify and calculate the mode such as principal component analysis.

## 5. Conclusions

This paper proposes a 3D indoor positioning method fused with the outputs of smartphone MEMS sensors for pedestrian positioning. A quaternion-based robust adaptive cubature Kalman filter (RACKF) algorithm is used to estimate the heading of pedestrians. The RACKF algorithm reduces the weight of stale data and adaptively modifies the model noise parameters based on MARG sensors. The fading memory weighting method and the limited memory weighting method are used to adaptively correct the statistical characteristics of the nonlinear system and reduce the estimation bias of the filter. An adaptive factor is based on prediction residual construction to overcome the Kalman filter model error and the influence of abnormal disturbance. Additionally, a robust M (maximum likelihood-type) estimator) is used to identify and control measurement outliers. The step size estimation is achieved by using the accelerometer data in the smartphone. The heading and step length of each step are optimized by the Kalman filter to reduce positioning error. The pitch angle, total accelerometer and barometer values of the smartphone are used to distinguish the pedestrian behavior patterns in the duration of effective step frequency. When upstairs and downstairs, according to the geometric information of the building stairs, the step length of pedestrians and the height difference of each step can be obtained. Combined with the differential barometric altimetry and step frequency detection method, the optimal height solution can be computed by the robust adaptive Kalman filter algorithm. In addition, based on the indoor map vector information, a heading calculation strategy of the 16-wind rose map is used to further improve the pedestrian positioning accuracy. Based on the movement law of pedestrians, the proposed 3D indoor positioning method effectively reduces the influence of sensor cumulative error on position calculation, and improves the positioning accuracy.

The experiments were conducted in an indoor environment conducted to verify the superiority of the proposed method. Height experimental results illustrate that the robust adaptive Kalman filter can improve height accuracy. Three-dimensional experimental results indicate that the proposed algorithm can provide more stable and accurate position estimation information. Therefore, the experimental results show that the proposed 3D indoor positioning method can provide an optimal model for pedestrian indoor location and navigation estimation. It is noticeable that, compared with other methods, the error of the proposed method is smaller and more stable. Therefore, it can be concluded that the proposed method can achieve better accuracy making it more suitable for indoor positioning with low-cost MEMS sensors of the smartphone.

In the future, we will focus on enhancing filter performance to improve the accuracy of the position. Moreover, different carrying modes are the key points of our research. For pedestrian elevator mode, identifying the pedestrian movement state and the change of barometer will be explored.

## Figures and Tables

**Table 1:** Detailed information of all participants (*S* is the step length parameter).

Participant	Sex	Height (m)	Weight (kg)	*S*
1	Male	1.78	60	0.48
2	Male	1.75	80	0.46
3	Female	1.72	81	0.49

**Table 2:** Statistical results of height error in the test (m).

Participant	Error Metrics	Differential Barometric Altimetry	Step Frequency Detection	RAKF
First	RMSE	0.4485	0.3404	0.2008
Second	RMSE	0.3428	0.2963	0.2723
Third	RMSE	0.2559	0.1799	0.1665

**Table 3:** Statistical results of the RMSE of location results (m).

Participant	Error Metrics	2D PDR	3D PDR	The Proposed Method
First	RMSE	2.6407	1.6514	1.0449
Second	RMSE	2.4968	1.5403	1.2663
Third	RMSE	3.2703	2.2312	1.6458

## Data Availability

Not applicable.

## Abbreviations

PDR	pedestrian dead reckoning
LBS	location-based services
MEMS	micro-electro-mechanical systems
RACKF	robust adaptive cubature Kalman filter
MARG	magnetic, angular rate, and gravity
RAKF	robust adaptive Kalman filter
RMSE	Root Mean Squared Error
GNSS	Global Navigation Satellite System
UWB	Ultra-Wideband
RFID	radio-frequency identification
CF	complementary filter
KF	Kalman filter
EKF	Extended Kalman filter
UKF	unscented Kalman filter
CKF	cubature Kalman filter
M-estimator	maximum likelihood-type estimator
FSM	finite-state machine
GPS	Global Positioning System
2D PDR	Two dimension pedestrian dead reckoning
3D PDR	Three dimension pedestrian dead reckoning
